# Agentic AI and Large Language Models in Radiology: Opportunities and Hallucination Challenges

**DOI:** 10.3390/bioengineering12121303

**Published:** 2025-11-26

**Authors:** Sara Salehi, Yashbir Singh, Kelly K. Horst, Quincy A. Hathaway, Bradley J. Erickson

**Affiliations:** 1 Radiology Informatics Lab, Department of Radiology, Mayo Clinic, Rochester, MN 55905, USA; horst.kelly@mayo.edu; 2 Department of Radiology, Mayo Clinic, Rochester, MN 55905, USA; singh.yashbir@mayo.edu; 3 Department of Radiology, University of Pennsylvania, Philadelphia, PA 19104, USA; quincy.hathaway@pennmedicine.upenn.edu

**Keywords:** agentic AI, large language models, radiology, hallucination, multi-agent systems, retrieval-augmented generation, medical imaging, clinical decision support

## Abstract

The field of radiology is experiencing rapid adoption of large language models (LLMs), yet their tendency to generate hallucinations (plausible but incorrect information) remains a significant barrier to trust. This comprehensive review evaluates emerging agentic artificial intelligence (AI) approaches, including multi-agent role-based systems, retrieval-augmented generation (RAG), and uncertainty quantification, to assess their potential for reducing hallucinations in radiology workflows. Evidence from 2024 to 2025 demonstrates that agentic AI can improve diagnostic accuracy and reduce error rates, though these methods remain computationally demanding and lack comprehensive clinical validation. Multi-agent frameworks enable cross-validation through role-based specialization and systematic workflow orchestration, while RAG strategies enhance accuracy by grounding responses in verified medical literature. Within multi-agent systems, uncertainty quantification enables agents to communicate confidence levels to one another, allowing them to appropriately weigh each other’s contributions during collaborative analysis. While multi-agent frameworks and RAG strategies show significant promise, practical deployment will require careful integration with human oversight, robust evaluation metrics tailored to medical imaging tasks, and regulatory adaptation to ensure safe clinical use in diverse patient populations and imaging modalities.

## 1. Introduction

In radiology departments worldwide, artificial intelligence integration has accelerated from 30% adoption in 2020 to over 75% in 2024, with large language models (LLMs) emerging as critical tools for report generation, image analysis, and clinical decision support [1]. Radiologists now routinely employ LLMs for structured reporting, automatic measurement extraction, and differential diagnosis generation, with studies showing 40% time reduction in report creation and 25% improvement in diagnostic consistency [2,3]. However, a critical barrier remains: LLM hallucinations in medical imaging contexts occur at rates of 8–15% across current systems —defined as AI-generated outputs that appear plausible but contain factually incorrect or fabricated medical information, generating plausible but incorrect anatomical descriptions, phantom lesions, or mischaracterized pathologies that directly threaten patient safety [4,5].

The research gap is precise: while detection methods identify hallucinations post-generation and training improvements reduce specific error types, no comprehensive framework prevents the underlying reasoning failures causing medical hallucinations [6,7]. Current single-agent LLMs process all cognitive tasks internally without systematic verification, leading to cascading errors, particularly dangerous in radiology, where subtle imaging features determine treatment decisions [8]. Vision-Language Models (VLMs), which analyze medical images alongside text, compound this challenge through dual-modal hallucinations—errors in visual interpretation propagate through textual generation, creating confident but incorrect reports indistinguishable from accurate ones without expert review [9,10].

This error rate raises important questions about whether these systems can be trusted for routine clinical use. Traditional approaches to addressing LLM hallucinations have focused primarily on detecting errors after they occur or training models on better data. However, a newer approach called “agentic AI” offers a different strategy. Agentic AI builds upon LLM technology but fundamentally changes how these models are deployed: instead of using a single LLM to handle all aspects of a task, agentic AI systems coordinate multiple LLM-based agents, each specialized for a distinct role in the workflow—such as information retrieval, summarization, analysis, and quality control. This architectural approach enables optimization of the LLM for each subtask while creating systematic checkpoints where errors can be detected through cross-validation between agents. This paper addresses three fundamental questions: What causes LLMs to hallucinate in medical contexts? Can agentic (multiple agents) approaches reduce hallucinations more effectively than single-agent systems? Are current agentic AI systems ready for deployment in real clinical settings?

## 2. Understanding LLM Hallucination in Radiology

We searched PubMed, arXiv, and IEEE Xplore (November 2023–November 2025) using terms “LLM hallucination”, “agentic AI”, “multi-agent”, combined with “radiology” or “medical imaging”. Included: peer-reviewed papers and preprints on hallucination mitigation. Excluded: non-medical applications, opinion pieces without data.

Throughout this review, we use “LLM” as an umbrella term encompassing both traditional Large Language Models that process just textual data and VLMs which process both images and text. VLMs face unique hallucination challenges beyond those of text-only LLMs. The process of translating visual medical information into textual descriptions introduces additional opportunities for error, particularly when subtle visual features must be converted into precise clinical language. The multimodal nature of VLMs requiring both accurate image interpretation and appropriate linguistic expression compounds the hallucination risk, as errors can occur in either the visual analysis stage or the text generation stage. In medical reporting, hallucinations manifest either false findings or as omission of essential information, both presenting notable risks for medical care [6].

**Figure 1:**
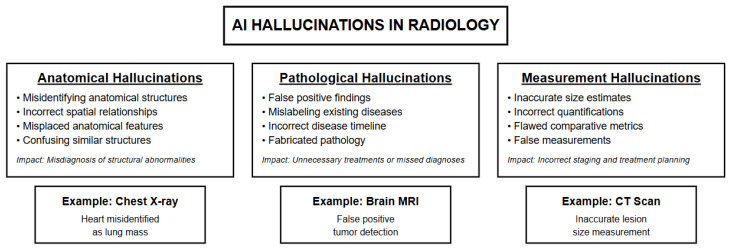
Taxonomy of AI hallucinations in medical imaging: anatomical, pathological, and measurement errors.

Recent research has systematically documented hallucination patterns across medical imaging applications, covering both visual image interpretation errors and associated textual reporting inaccuracies, providing a comprehensive taxonomy for understanding these errors. The study divides hallucinations into three categories: anatomical, pathological, and measurement-based [4]. Anatomical hallucinations include misidentifying structures, misplacing anatomical features, or misrepresenting spatial relationships. Pathological hallucinations encompass false positives, false negatives, mislabeling existing disease, or temporal misrepresentations such as describing acute findings as chronic or incorrectly staging disease progression. Measurement hallucinations involve both approximate size assessment errors (describing lesions as “large” versus “small”) and precise quantification mistakes (reporting “3.2 cm” when the actual measurement is “4.1 cm”), along with flawed comparative metrics [4] (Figure 1).

Hallucination quantification varies across studies, where some use binary detection (present/absent), others employ severity scales (minor/major/critical), and recent benchmarks like MedHallu provide standardized datasets with ground-truth annotations. Reported rates (8–15%) reflect this methodological heterogeneity, necessitating standardized metrics for meaningful comparison [10].

The frequency of LLM hallucinations in research settings and experimental clinical applications appears concerning based on emerging evidence. While overall diagnostic accuracy improved with AI assistance, and radiology residents achieved higher accuracy in differential diagnoses of brain MRI when assisted by LLMs compared to standard approaches [5], the presence of confident-sounding but incorrect information poses significant risks for clinical decision-making. This proves particularly problematic when AI systems fail to recognize or correctly estimate critical findings like midline shift in MRI, potentially resulting in reports that minimize serious conditions and create downstream risks for patient care [7].

Understanding the underlying causes of AI hallucinations is crucial for developing effective solutions. Medical images present complex, multi-layered information that challenges current AI systems in accurately interpreting subtle findings. These systems frequently confuse related but distinct medical terms or anatomical structures, particularly when visual or linguistic similarities exist [8]. AI models tend to generate associations between related concepts even when available evidence is limited or ambiguous. Most concerning is their tendency to produce plausible-sounding responses rather than appropriately acknowledging uncertainty when faced with ambiguous information, a particularly dangerous behavior in medical settings where admitting uncertainty would be both more appropriate and safer than providing potentially incorrect information.

## 3. Current Approaches to Address Hallucinations

While Section 2 categorized hallucination types, this section examines mitigation strategies currently deployed in clinical settings. Several strategies have emerged to address LLM hallucinations in radiology, each with distinct advantages and limitations. Technical improvements involve enhancing both prompt design and the text generation process, for example, through retrieval-augmented guidance or medically constrained decoding, to guarantee that output remains limited to the intended and clinically relevant content [7]. Detection methods have shown promise in identifying hallucinations after they occur. Researchers have developed sophisticated detection systems that can flag both individual hallucinated sentences and entire reports with high precision [9]. The introduction of MedHallu, the largest publicly available benchmark for medical hallucination, provides a valuable tool for evaluating LLMs and guiding their safe application in critical medical settings [10]. Additionally, MedHallBench represents a new benchmark designed to evaluate and reduce hallucinations in medical large language models [4]. The most successful approach documented so far comes from knowledge-based fixes using retrieval-augmented generation (RAG). RAG is a technique in which documents are encoded in a way that their concepts and content are retrievable using LLMs rather than requiring matches to specific words. Wada and colleagues used RAG to eliminate hallucinations in radiology contrast guidance. By grounding AI responses in documents on contrast material use, they reduced hallucination rates from 8% to 0% while maintaining response speed and protecting patient privacy [11]. Training improvements have also shown promise, with researchers using Direct Preference Optimization to reduce false reports of prior exams in chest X-ray analysis, achieving a 4.8-fold reduction in spurious mentions while maintaining clinical accuracy [12]. In Direct Preference Optimization, the output of an LLM is rated by humans and that feedback is used to improve the LLM response. While these approaches show promise, they mostly address hallucination detection or correction after the fact and do not necessarily prevent the underlying reasoning problems that cause hallucinations in the first place.

## 4. Agentic AI: Multi-Agent Approaches

**Figure 2:**
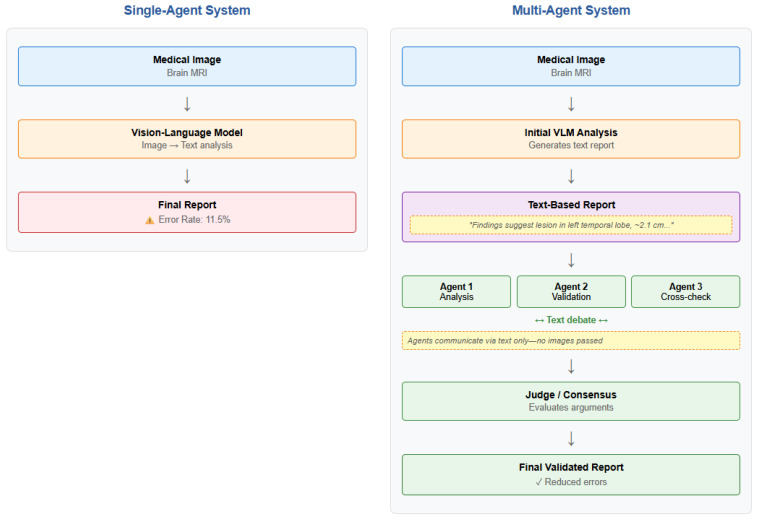
Comparison of single-agent (11.5% error) versus multi-agent architectures with specialized role distribution.

Agentic AI refers to systems where multiple LLM-based agents work together—distinct from multi-modal LLMs that process vision and language within a single model. While multi-modal LLMs handle multiple inputs internally, agentic systems distribute tasks across specialized agents, enabling external validation checkpoints between processing stages—collaboratively to solve complex problems. Each agent is an LLM, and each is assigned a specialized role in a structured workflow. Agentic AI employs multiple LLM-based agents with distinct responsibilities: one might search for relevant information, another summarizes those documents, others perform analysis from different perspectives, and a final agent evaluates quality. A potential advantage compared to a single LLM that can be exceedingly difficult to understand is that agents communicate with each other using natural language text, making the reasoning process more transparent and interpretable. Madrid-García and colleagues argue that while individual LLMs lack the “slow-thinking and divergent-thinking” abilities needed for reliable medical reasoning, distributing tasks across multiple LLM-based agents with separate, specialized roles can address these fundamental limitations [1] (Figure 2).

**Figure 3:**
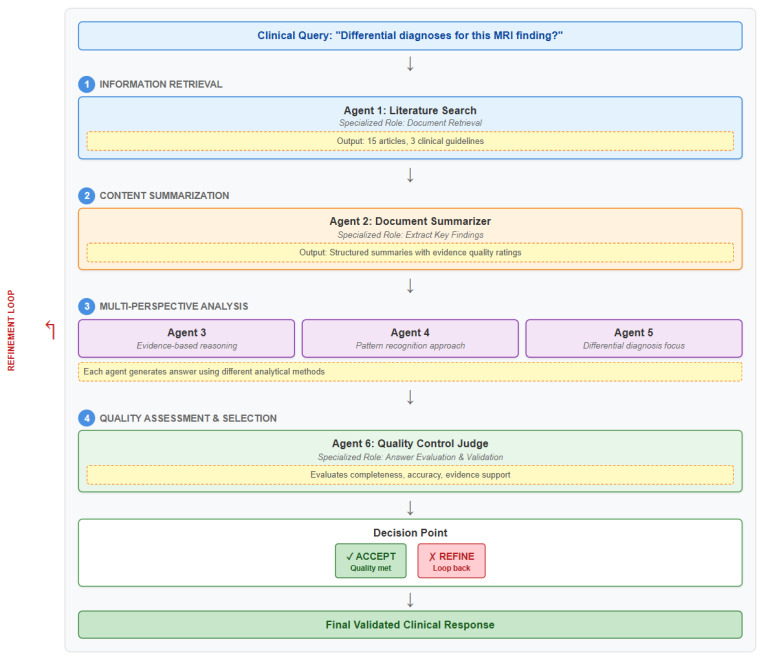
Role-based multi-agent workflow: literature search, summarization, independent analysis, and quality control stages. A key challenge in multi-agent systems is determining how agents should effectively communicate their confidence levels and uncertainty estimates. Yoffe and colleagues developed methods for AI agents to share not just their conclusions but also quantified measures of how certain they are about those conclusions [14]. They evaluated two main approaches for uncertainty communication: explicit confidence statements in text and attention-based methods that adjust how much agents consider each other’s input based on mathematical confidence levels. The attention-based approach performed significantly better, with improvements that increased as the uncertainty estimates became more dependable and precise.

The most effective multi-agent systems utilize role-based architectures where LLM-based agents with distinct, specialized roles collaborate through structured workflows to reach conclusions. Rather than having multiple agents perform similar analytical tasks such as all agents debating the same diagnostic question, which reasoning models can handle internally modern multi-agent systems assign each agent a specific function in a sequential process, creating a clear division of labor. For example, in a radiology consultation workflow: Agent 1 searches medical literature to find relevant documents addressing the clinical question; Agent 2 summarizes each retrieved document; Agents 3–5 independently develop potential answers based on these summaries, each applying different reasoning approaches; and Agent 6 evaluates the proposed answers, selecting the best response or, if quality is insufficient, triggering the process to restart with refined search criteria. This role-based specialization allows each agent to focus on a discrete task—information retrieval, summarization, analysis, or quality assessment creating a clear division of labor that distinguishes multi-agent approaches from both single LLMs that attempt all functions internally and from debate-based multi-agent systems where all agents perform similar analytical tasks. Lin and colleagues demonstrated role differentiation in medical image interpretation, where agents assumed distinct analytical perspectives rather than all performing identical analyses [8]. Sun and colleagues advanced this approach by using mathematical models called Markov Chains to structure the interaction process more rigorously. Their system transitions between diverse types of agent interactions based on confidence levels, showing significant improvements over traditional single-agent systems in both accuracy and reliability [13] (Figure 3).

## 5. Mathematical Foundations and Mechanisms of Action

Recent theoretical work has provided formal frameworks for understanding hallucinations in language models. Kalai and colleagues define hallucination as the production of an invalid answer in cases where at least one valid answer exists [15]. They establish a mathematical relationship showing that hallucination rates are fundamentally bound by classification errors, leading to inequalities demonstrating that hallucination is mathematically inevitable whenever the underlying classifier has non-zero errors. Their analysis reveals that reinforcement learning from human feedback can exacerbate hallucinations because it penalizes uncertainty and encourages models to output confident responses, even when incorrect. Unlike single LLMs that perform all cognitive tasks internally, multi-agent systems address hallucinations through role-based specialization and workflow orchestration, leveraging multiple LLM-based agents that are each optimized for specific subtasks. The key advantage lies in dividing complex medical tasks into discrete, specialized subtasks, each managed by an agent optimized for that specific function. Task decomposition allows complex medical queries to be broken into manageable components: information retrieval, summarization, analysis, and quality control with each agent focusing exclusively on its specialized role. Specialized agent design enables optimization of individual agents for specific functions; for example, a literature search agent can be fine-tuned on medical database queries, while an analysis agent focuses on clinical reasoning. Sequential validation creates natural checkpoints where outputs from one agent are verified before becoming inputs to the next, reducing error propagation. Quality control mechanisms through resolute judge agents provide systematic evaluation of outputs against predefined clinical standards, with the ability to trigger workflow refinement when quality thresholds are not met. This architectural approach differs fundamentally from reasoning models that perform all cognitive steps internally multi-agent systems externalize and distribute these steps across specialized components with explicit handoffs and validation points.

## 6. Current Evidence and Clinical Applications

Recent research has demonstrated promising results for agentic AI approaches in specific applications. Wada and colleagues achieved remarkable success with their retrieval-augmented generation approach, with hallucinations detected in 8% of outputs from the base model completely absent (0%) when using the RAG-enhanced version (χ^2^(Yates) = 6.38, *p* = 0.012; Fisher *p* = 0.0068) [11]. Their approach maintained response speed while improving reliability, suggesting that some agentic approaches can meet clinical performance requirements.

Multi-agent role-based systems have shown consistent improvements over single-agent baselines across multiple studies. Sun and colleagues demonstrated that adopting a Markov chain-based multi-agent framework significantly improves accuracy in detecting hallucinations in LLMs [13]. Similarly, Yoffe and colleagues demonstrated that attention-based uncertainty communication outperformed simpler approaches, revealing that LLMs are capable of handling information beyond standard textual inputs [14]. However, these positive results come with important limitations. Most studies evaluate simple scenarios rather than complex, real-world clinical cases that represent the full challenge of clinical practice. The computational overhead of multi-agent systems has been documented but not thoroughly analyzed in terms of cost-effectiveness for clinical deployment. Additionally, the requirement for advanced language models limits the generalizability of current results, as many healthcare systems may not have access to the computational resources needed for effective agentic AI implementation (Table 1).

## 7. Limitations and Implementation Challenges

Agentic AI systems face several significant challenges that limit their immediate clinical deployment. Computational requirements represent a major barrier, as these systems require significantly more resources than single-agent approaches [15]. Running multiple AI models simultaneously and coordinating their interactions increases processing time and costs, which may limit practical deployment in healthcare settings where cost-effectiveness is a major consideration.

Limitation: Current benchmarks inadequately measure real-world computational costs across diverse hospital IT infrastructures [16]. Limitation: No standardized protocols exist for evaluating multi-agent systems against single-agent baselines in prospective trials [17].

Model dependencies present another challenge, as current research shows that effective agentic systems require powerful, state-of-the-art language models to function properly. Lin and colleagues observed that most current open-source models lacked the capacity for effective multi-agent collaboration, with some unable to follow complex prompts while others failed to maintain their specialized roles during agent interactions [8]. This requirement for advanced models may limit accessibility and significantly increase operational costs. Limitation: Proprietary model requirements create vendor lock-in and limit reproducibility across institutions [18].

Limited clinical testing represents a critical gap, as most current research consists of laboratory studies and controlled experiments rather than rigorous clinical trials in real-world settings. Without extensive clinical validation, it is difficult to assess whether these systems are ready for routine medical use and whether they will maintain their effectiveness in complex clinical environments.

**Figure 4:**
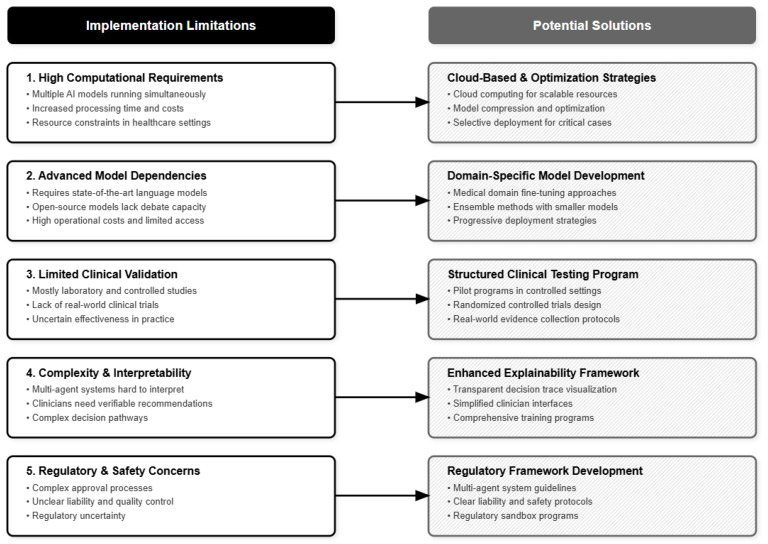
Five implementation barriers and corresponding solutions for clinical deployment.

The complexity of multi-agent systems may also make it harder to interpret than simpler single-agent approaches, which could be problematic for clinical settings where healthcare providers need to understand and verify AI recommendations (Figure 4). Limitation: Black-box agent interactions may violate regulatory requirements for explainable medical AI [19].

Regulatory and safety concerns add another layer of complexity, as the multi-agent nature of these systems complicates traditional medical device regulation and approval processes. Questions about liability, safety validation, and quality control become more complex when multiple AI agents participate in medical decisions, creating regulatory uncertainty that may slow adoption. Responsibility assignment in multi-agent errors requires hierarchical accountability: the supervising radiologist maintains primary liability, while technical responsibility traces through agent logs documenting each decision point. Proposed frameworks include mandatory audit trails showing agent contributions, confidence scores, and decision rationales for post hoc analysis [19].

## 8. Future Directions and Clinical Translation

Based on current evidence, agentic AI appears most suitable for specific, well-defined tasks rather than general clinical decision-making. Applications like quality assurance, second-opinion generation, or research assistance may be appropriate starting points for implementation. The most promising near-term approach involves human-AI collaboration where agentic systems augment rather than replace human expertise, allowing healthcare providers to benefit from improved AI reliability while maintaining appropriate oversight and final decision-making authority. Several research priorities need immediate attention before widespread clinical deployment becomes feasible. Clinical validation requires prospective protocols including: (1) parallel reading studies with 100+ consecutive cases across modalities, (2) randomized controlled trials comparing agentic-assisted versus standard reporting, (3) longitudinal tracking of diagnostic accuracy and patient outcomes, (4) assessment in emergency and routine settings with diverse pathology prevalence, including real-world clinical settings and patient outcomes rather than just accuracy metrics measured in laboratory conditions. Cost-effectiveness analysis of the computational overhead requires careful economic evaluation, as healthcare systems need unambiguous evidence that improved accuracy justifies increased costs. Comprehensive testing of edge cases and failure modes is necessary for understanding when and how agentic systems fail, which is crucial for safe clinical deployment and appropriate risk management.

Critical gaps remain unaddressed in current research [17]. First, no studies evaluate agentic AI performance across diverse imaging modalities (CT, MRI, ultrasound, nuclear medicine) simultaneously [20]. Second, interaction effects between multiple agents remain poorly understood—does increasing agent count beyond five improve or degrade performance? Third, failure mode analysis is absent; we lack understanding of catastrophic failure scenarios where all agents agree on incorrect diagnoses. Fourth, pediatric and rare disease applications remain untested, where training data scarcity may fundamentally limit multi-agent effectiveness. Future studies must address: (1) cross-modality generalization [20,21,22], (2) optimal agent architectures for specific clinical tasks, (3) human-AI interaction patterns in time-pressured emergency settings, and (4) long-term learning capabilities where agents improve through clinical deployment [21,23,24,25].

The field needs standardization through agreed-upon evaluation metrics, safety standards, and best practices for agentic AI development and deployment to ensure consistent quality and safety. Appropriate use of LLMs in clinical settings requires adequate training and familiarity with their nuances [22]. For organizations considering implementation, recommendations include starting with low-risk applications where errors have limited consequences, maintaining human oversight and final decision authority, implementing comprehensive uncertainty quantification and communication, conducting thorough local validation before deployment, and developing clear protocols for handling AI disagreements or failures [23].

## 9. Conclusions and Clinical Implications

Agentic AI demonstrates potential for reducing LLM hallucinations in radiology through multi-agent architectures and retrieval-augmented generation. Evidence suggests 0–42% error reduction in controlled settings, though clinical validation remains limited. Near-term deployment appears feasible for quality assurance and second-opinion generation under human supervision. However, computational costs, model dependencies, and regulatory uncertainties constrain immediate adoption. Future priorities include standardized evaluation metrics, prospective clinical trials, and cost-effectiveness analyses. While promising, agentic AI currently supplements rather than replaces radiologist expertise.

## Figures and Tables

**Table 1:** Summary of Agentic AI Approaches and Outcomes.

Method	Study	Hallucination Reduction	Key Finding	Limitation
RAG	Wada et al. [11]	8% → 0%	Complete elimination in contrast guidance	Domain-specific only
Multi-agent debate	Lin et al. [8]	35% improvement	Role differentiation crucial	Requires advanced models
Markov chain framework	Sun et al. [13]	42% accuracy gain	Confidence-based transitions	High computational cost
Uncertainty quantification	Yoffe et al. [14]	28% better calibration	Attention-based outperforms text	Complex implementation
Direct Preference Optimization	Banerjee et al. [12]	4.8-fold reduction	Spurious exam mentions eliminated	Limited to specific errors

## Data Availability

No new data were created or analyzed in this study.
